# The future for the therapeutics of abdominal aortic aneurysm: engineered nanoparticles drug delivery for abdominal aortic aneurysm

**DOI:** 10.3389/fbioe.2023.1324406

**Published:** 2024-01-05

**Authors:** Pengchong Du, Yachen Hou, Chang Su, Jiamin Gao, Yu Yang, Jinying Zhang, Xiaolin Cui, Junnan Tang

**Affiliations:** ^1^ Department of Cardiology, The First Affiliated Hospital of Zhengzhou University, Zhengzhou, China; ^2^ Henan Province Key Laboratory of Cardiac Injury and Repair, Zhengzhou, China; ^3^ Henan Province Clinical Research Center for Cardiovascular Diseases, Zhengzhou, China; ^4^ School of Medicine, The Chinese University of Hong Kong, Shenzhen, China

**Keywords:** abdominal aortic aneurysm, engineered nanoparticles, screening, extracellular vesicles, treatment

## Abstract

Abdominal aortic aneurysm (AAA) is a severe cardiovascular disease with a high mortality rate. Several screening and diagnostic methods have been developed for AAA early diagnosis. Open surgery and endovascular aortic repair (EVAR) are clinically available for patients who meet the indications for surgery. However, for non-surgical patients, limited drugs exist to inhibit or reverse the progression of aneurysms due to the complex pathogenesis and biological structure of AAA, failing to accumulate precisely on the lesion to achieve sufficient concentrations. The recently developed nanotechnology offers a new strategy to address this problem by developing drug-carrying nanoparticles with enhanced water solubility and targeting capacity, prolonged duration, and reduced side effects. Despite the rising popularity, limited literature is available to highlight the progression of the field. Herein, in this review, we first discuss the pathogenesis of AAA, the methods of diagnosis and treatment that have been applied clinically, followed by the review of research progressions of constructing different drug-loaded nanoparticles for AAA treatment using engineered nanoparticles. In addition, the feasibility of extracellular vesicles (EVs) and EVs-based nanotechnology for AAA treatment in recent years are highlighted, together with the future perspective. We hope this review will provide a clear picture for the scientists and clinicians to find a new solution for AAA clinical management.

## 1 Introduction

The aorta, a sizable blood vessel originating from the heart, exhibits elasticity and undergoes expansion and contraction with each cardiac cycle. If the structural integrity of the aortic wall deteriorates over time, the potential for an aneurysm to develop arises (Baman and Eskandari, 2022). Specifically, aneurysms located in the infrarenal segment of the aorta, measuring at least 1.5 times the diameter of a healthy aorta or exceeding 30 mm in diameter, are classified as abdominal aortic aneurysms (AAA) (Golledge, 2019; Debono et al., 2021). AAA, a severe cardiovascular disease, resulted in 167,200 deaths worldwide in 2017, and 3 million disability-adjusted life years attributed to AAA (Golledge et al., 2023). However, the accuracy of statistical data is affected by the low rates of post-mortems. Nevertheless, these statistics can still serve as valuable references (Virani et al., 2020; Golledge et al., 2023). Notably, the elderly population is particularly susceptible to AAA development. Moreover, when considering gender, the risk of AAA is significantly higher in males (4%–8%) compared to females (0.5%–1.5%) (Klink et al., 2011; Schanzer and Oderich, 2021). Furthermore, prevalence rates of AAA among Black and Asian individuals are lower than those among White individuals (Chan et al., 2020; Schanzer and Oderich, 2021; Summers et al., 2021). Many pathologic factors cause degradation and dilation of the abdominal aortic vessel wall, including elevated matrix metalloproteinase (MMP) (Nosoudi et al., 2015; Bai et al., 2022), degradation of the extracellular matrix (ECM) (Rabkin, 2017), infiltration of inflammatory cells (Schmitz-Rixen et al., 2016), calcification of the vessel wall (Zimmermann, 2018), apoptosis of vascular smooth muscle cells (VSMCs) and oxidative stress (Schmitz-Rixen et al., 2016; Sanchez-Infantes et al., 2021), have been identified contributing to the development and progression of AAA.

The increased size of the aneurysm increases the risk of rupture. Therefore, early screening assumes paramount significance, leading countries like Sweden, the US, and the UK to implement ultrasound screening for high-risk populations (Golledge et al., 2023). In the initial stages, most patients with abdominal aortic aneurysms (AAA) remain undiagnosed until the manifestation of notable clinical indicators, indicating imminent rupture and necessitating elective surgical interventions such as open surgery and EVAR. Elective surgery remains the only option available for the treatment of AAA (Quaye et al., 2022). Based on the guidelines, a patient with an aortic diameter of ≥55 mm (male) or ≥50 mm (female) is suitable for elective surgery (Powell et al., 2007). However, in the case of patients with smaller diameters of AAA, surgical intervention does not necessarily lead to a higher rate of survival and may instead exacerbate the medical and psychological burden on the patient (Toczek et al., 2016). Clinical evidence supports the notion that smoking cessation and effective management of hypertension can be advantageous in delaying the growth and rupture of aneurysms (Sweeting et al., 2012; Benson et al., 2018). Conversely, the use of β-blockers and other antihypertensive medications may aid in reducing cardiovascular risk but have limited efficacy in impeding, inhibiting, or reversing the progression of AAA (Wang et al., 2018; Zhang et al., 2018; Schanzer and Oderich, 2021). This may be related to the formation of multiple layers of thrombus within the pseudolumen, which can prevent the target and release of drugs (Sivaraman et al., 2016).

AAA is induced by a combination of factors that could be targeted therapeutically. Experimental studies performed in small animal studies have shown effectiveness in inhibiting the progression of AAA (Sivaraman et al., 2016; Rabkin, 2017; Senemaud et al., 2017; Yoshimura et al., 2018; Bai et al., 2022). This inhibition was achieved through pathways that inhibit inflammatory cells, modulate the renin-angiotensin system, and reduce ECM degradation via MMP inhibitors. Recent studies have also revealed that enterobacteria and their metabolite butyric acid significantly attenuate AAA progression by reducing aortic wall neutrophil infiltration and NET formation in AAA patients, providing new insights into AAA clinical management (Tian et al., 2022). However, traditional routes of administration have been impacted by inadequate targeting capacity and side effects. As a result, an effective delivery vehicle is of utmost importance (Golledge et al., 2017).

The recent advances in engineered nanoparticles offer new strategies for achieving drug delivery. Nanoparticles, which are particles ranging from 1 to 100 nm in size and much smaller than human cells (Suri et al., 2007; Zhu et al., 2020), are functionally diverse with superior biocompatibility and enhanced permeability. They possess long-circulating potential, which is ideal for long-term therapeutics (Chen et al., 2022). However, it is important to note that engineered nanoparticles constructed too small are easily cleared from the body, have low drug loading, and some materials are toxic. Thus, optimizing the material and size of nanoparticles can improve cellular uptake while avoiding removal by the reticuloendothelial system. The surface modification enables nanoparticles to bind with specific cell receptors, achieving targeted delivery (Hoshyar et al., 2016; Jiang et al., 2017; Zhu et al., 2020). In recent years, research has widely explored engineered nanoparticle drug delivery technologies for the treatment of AAA with encouraging results. This paper summarizes the current status of constructing different drug-loaded nanoparticles to treat AAA using tissue-engineered technology. Additionally, it illustrates the feasibility of emerging EVs and EVs-based nanotechnology for the treatment of AAA. Finally, we discuss the potential application of these new technologies in AAA treatment, shedding light on a new direction for AAA clinical management.

## 2 The pathogenesis of AAA

Early research indicated that atherosclerosis plays a pivotal role in the pathogenesis of AAA (Anagnostakos and Lal, 2021). Various methodologies have been employed to investigate the pathogenesis of AAA patients, encompassing the analysis of risk factors associated with AAA diagnosis and progression in population-based screening studies, case-control inquiries, and cohort investigations, as well as the comparison of blood and tissue samples obtained from AAA patients and healthy populations (Golledge, 2019). In addition, risk factors such as age, smoking, race, and gender have a significant effect on the incidence of AAA (Salem et al., 2009; Ullery et al., 2018; Anagnostakos and Lal, 2021). It is mainly believed that AAA is an acquired, immune-driven destruction of the aortic wall (Golledge, 2019). This complex process involves several potential mechanisms that lead to the onset and progression of human disease, and there are three main types: the apoptosis of smooth muscle cells in the vessel wall, infiltration of immune cells, along the disruption and remodeling of the ECM structure (Quintana and Taylor, 2019; Weaver et al., 2022). Recent years have witnessed an increasing amount of evidence that substantiates this perspective (Weaver et al., 2022).

**FIGURE 1:**
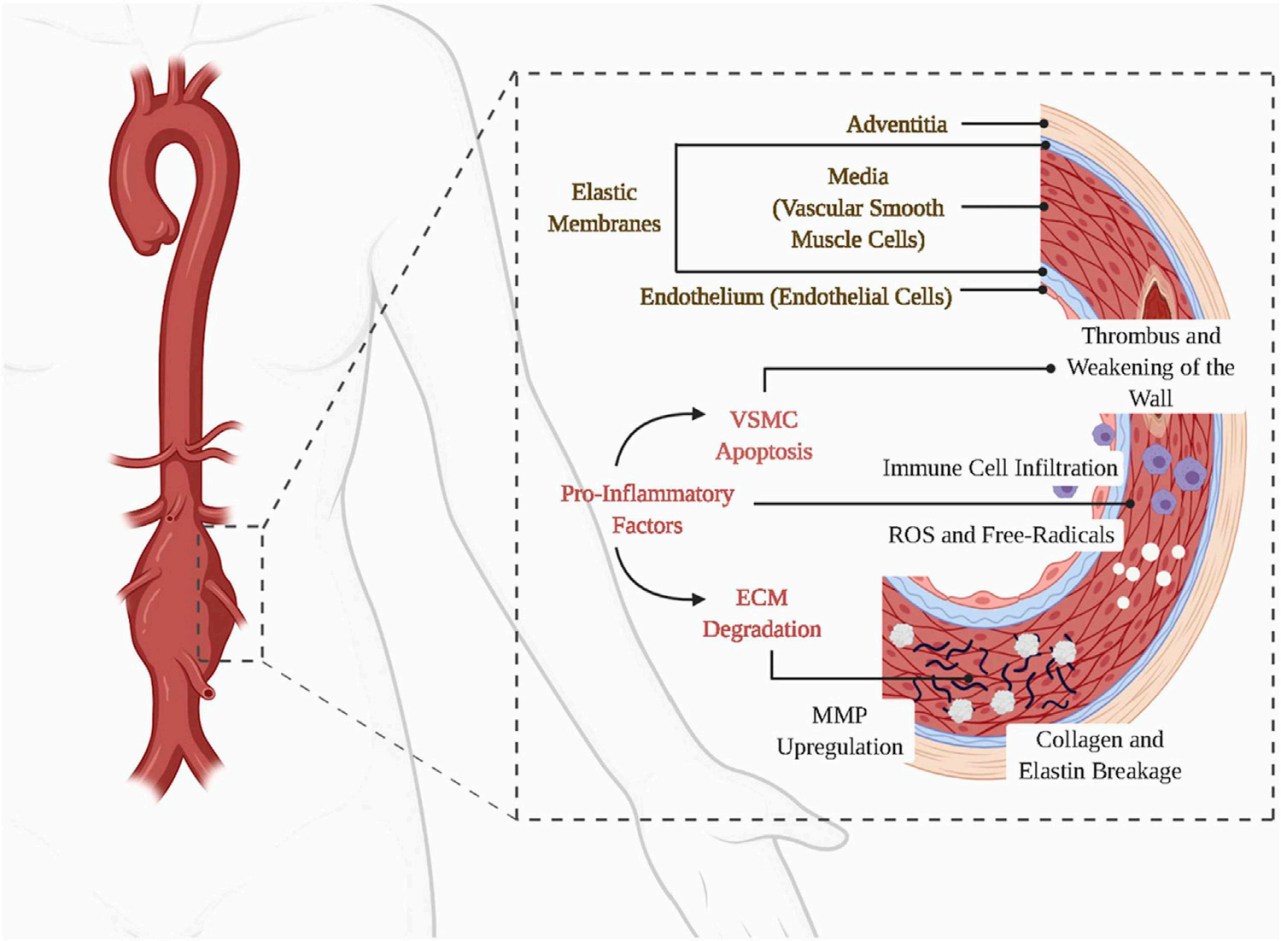
Etiology and pathogenesis of AAA. Panel 1 reproduced from Ref. (Weaver et al., 2022). Copyright 2022 Elsevier.

The normal vascular wall of the abdominal aorta is divided into three layers, with the intima formed by endothelial cells and connective tissue, and the media and externa consisting of a complex structure of the ECM, VSMCs and various vascular cells (fibroblasts, etc.) (Li et al., 2022). When arterial damage occurs, particularly affecting the media of the vessel wall, various immune cells such as macrophages (the main infiltrating cell type in the diseased vessel wall), neutrophils, B-cells, and T-cells infiltrate the affected region. This infiltration results in the release of proinflammatory cytokines and reactive oxygen species (ROSs), which subsequently promote the production of MMP and other proteases. Consequently, elastin, collagen (specifically types I and III), matrix cellular proteins, and numerous other proteins within the extracellular matrix (ECM) undergo degradation (Sakalihasan et al., 2018). This phenomenon initiates the disruption of the mid-aortic layer, apoptosis, and dysfunction of VSMCs, as well as the accumulation of additional inflammatory cells. Consequently, a detrimental cycle ensues, resulting in the gradual thinning and weakening of the vessel wall, ultimately culminating in the development of an aneurysm (Figure 1) (Davies, 1998; Nordon et al., 2011; Anagnostakos and Lal, 2021; Dang et al., 2022; Weaver et al., 2022).

In all commonly used rodent models of AAA, the presence of macrophages, neutrophils, B-cells, and T-cells has been observed (Rateri et al., 2011; Alsiraj et al., 2017; Lareyre et al., 2017; Yu et al., 2017; Coscas et al., 2018). Consequently, the downregulation of immune-mediated aortic destruction emerges as a crucial objective to restrict the progression of AAA. Moreover, in human samples of AAA obtained during open surgical repair, a diverse array of natural and acquired immune cells and their products are found within the aortic wall and intraluminal thrombus, providing additional evidence in favor of the inflammation-mediated theory of AAA (Lindeman et al., 2011; Koole et al., 2012; Biros et al., 2014; Zhou et al., 2015). It has also been shown that angiotensin I/II receptors in the aorta are involved in the formation and progression of AAA (Anagnostakos and Lal, 2021). This has potential clinical implications. Presently, numerous studies are being conducted to assess the efficacy of interventions such as doxycycline, tegretol, angiotensinogen-converting enzyme inhibitors (ACEI), and angiotensin receptor blockers (ARB) in mitigating the occurrence and halting the progression of AAA (Anagnostakos and Lal, 2021).

## 3 Screening and clinical diagnosis of AAA

Most people with AAA are asymptomatic and their condition is only detected through relevant imaging tests for other disease diagnoses or incidentally discovered during regular health checks. As rupture of aneurysms results in a very high mortality rate, early screening is crucial to reduce mortality (Oliver-Williams et al., 2018; Sprynger et al., 2019). Ultrasound is recommended as the imaging modality of choice to screen patients with AAA (Chaikof et al., 2018). For individuals with an aneurysm diameter between 30 mm and 39 mm, ultrasound screening is recommended every 3 years. For those with a size between 40 mm and 49 mm, screening should be done annually, while those with an AAA size between 50 mm and 55 mm require screening every 6 months (Table 1) (Chaikof et al., 2018; Sprynger et al., 2019; AORN J, 2020).

**TABLE 1:** Guidelines for ultrasonic screening of AAA.

Diameter of AAA	Screening time
30mm–39 mm	Every 3 years
40mm–49 mm	Every 1 year
50mm–55 mm	Every 6 months

*AAA, abdominal aortic aneurysms.

The typical AAA patient usually has several risk factors, including family history, being a long-term smoker, being an older male, history of other large vessel aneurysms, and comorbidities (e.g., hypertension, hypercholesterolemia, atherosclerosis) (Ullery et al., 2018). These patients may present symptoms such as abdominal pain, low back pain, back pain, and a pulsating abdominal mass, caused by the aneurysm compressing the surrounding peritoneum and organs. In severe cases, patients may present to the emergency department with hypotension and shock due to AAA rupture. However, not all patients are symptomatic, and some symptoms may present in patients with other diseases (e.g., acute myocardial infarction, kidney stones, gastrointestinal disorders) (Moxon et al., 2010). Precise diagnosis of AAA based on physical symptoms remains a clinical challenge. Patients with suspected AAA require early diagnosis confirmation of the diagnosis. Digital Subtraction Angiography (DSA) is one of the most important imaging methods for diagnosing AAA. DSA imaging has a high accuracy for staging results and morphologic determination of AAA and also allows for intravascular therapeutic manipulation, which is not available with other imaging examinations. However, DSA is an invasive test and is not as fast as computed tomography angiography (CTA), which is first chosen clinically to accurately assess intravascular structures. Recently, CTA has also been combined with artificial intelligence, allowing for automated software analysis, enabling rapid quantitative analysis of intraluminal thrombosis and calcification (Lareyre et al., 2021). Magnetic resonance angiography (MRA) is another method of evaluating AAA that is not usually performed in emergencies and can be used as an adjunct to imaging in patients with contraindications to CT contrast (e.g., renal insufficiency and allergies) (Howell and Rabener, 2016; Chaikof et al., 2018).

## 4 Current clinical management of AAA

### 4.1 Surgical treatment

Patients with symptomatic AAA and asymptomatic patients with an aneurysm size of ≥55 mm in men and ≥50 mm in women require surgical intervention (Wang et al., 2018). Surgical options for AAA include open repair surgery and EVAR. Open repair surgery is performed under general anesthesia by making a suitable abdominal incision, usually along the anterior midline to expose the aortic aneurysm. The aneurysm is then longitudinally incised to remove the thrombus, followed by implanting an arterial prosthesis into the aorta. EVAR, on the other hand, is performed under local anesthesia, where a stent graft is implanted through the femoral artery into the lesion to close the aneurysm and prevent rupture (Tsilimparis et al., 2017; Chaikof et al., 2018; Anagnostakos and Lal, 2021). In most cases, EVAR is the preferred option for AAA patients. For patients whose vascular anatomy does not meet the requirements of EVAR, open repair surgery can still be performed (Williamson and Babrowski, 2018). Additionally, open repair surgery is often used in cases of intra-aneurysmal leakage or severe graft infection after EVAR (Qrareya and Zuhaili, 2021).

One study compared EVAR with unrepaired patients who were not eligible for open repair and found that EVAR did not improve life expectancy (Sweeting et al., 2017). Several studies have also compared open repair surgery with EVAR in a randomized fashion and found that survival rates in patients with early EVAR are higher than in open repair surgery, but this advantage diminished after 4 years. In the 15-year follow-up, survival rates in patients who underwent EVAR were found to be much lower due to reoperation and secondary aneurysm rupture (Patel et al., 2016). Open surgery is highly invasive and not suitable for some patients with advanced age and severe underlying disease, resulting in higher postoperative complications and mortality. In the long term, EVAR needs to further improve its durability to increase patient survival (Haque and Bhargava, 2022).

### 4.2 Medication

The lack of effective drugs for AAA treatment remains a tremendous clinical challenge. The β-adrenergic receptor blocker propranolol was the first drug to treat AAAs in animal models. However, it fails to slow AAA growth in subsequent randomized controlled trials (Propanolol Aneurysm Trial, 2002; Yoshimura et al., 2018). In addition, a few meta-analyses found that statins slow the progression of atherosclerosis, affecting AAA growth and reducing the likelihood of aneurysm rupture (Takagi et al., 2012; Salata et al., 2018; Haque and Bhargava, 2022). Some researchers found a high detection rate of *Chlamydia* pneumonia at the lesion site in AAA patients and therefore suspected that AAA progression may be related to it. However, the antibiotic treatment did not significantly reduce AAA growth (Juvonen et al., 1997; Yoshimura et al., 2018). Doxycycline, an MMP inhibitor, has not been clinically shown to have a therapeutic effect on AAA growth in randomized testing (Meijer et al., 2013). Anti-hypertensive drugs are more likely to maintain the patient’s blood pressure within a reasonable range, preventing the aneurysm from rupturing. However, some randomized trials indicated that anti-hypertensive drugs are ineffective in treating AAA (Bicknell et al., 2016). A similar conclusion was drawn from the randomized trial of the mast cell inhibitor (Sillesen et al., 2015).

## 5 Animal models of AAA

At present, the animal models of AAA are mainly mice and rats. The common methods to induce AAA formation include elastase perfusion, angiotensin II infusion, and calcium chloride (CaCl_2_) application.

### 5.1 Elastase perfusion

Elastase perfusion is widely employed in the establishment of rodent models for AAA. This intricate procedure necessitates accessing the abdominal cavity and administering elastase via local injection into the infrarenal segment of the abdominal aorta (Busch et al., 2016; Yu et al., 2017; Busch et al., 2018). An obvious advantage of this model is that aneurysm formation occurs only in the portion of the aorta injected with elastase which would result in dilatation of the entire aortic wall. Another advantage is that this model allows for the observation of macrophage infiltration and heightened MMP expression within the aortic wall, closely resembling human pathology (Alan and Cassis, 2004). The limitations of this model are that the surgical procedure is difficult, involves acute injury (the time to form AAA is short), is not suitable for studies of long-term inhibition of AAA growth by drugs, and the size and severity of AAA formed varies greatly (Rd et al., 2001; Wang et al., 2014).

### 5.2 Angiotensin II infusion

The induction of the AAA model through the injection of angiotensin II is another frequently employed technique (Trachet et al., 2016; Alsiraj et al., 2017). In contrast to the elastase perfusion model, the surgical procedure of this model is easy, allowing for the long-term induction of AAA through the subcutaneous implantation of an osmotic pump containing angiotensin II. Furthermore, the aortic wall exhibits inflammatory cell infiltration, lumen dilatation, and ECM degradation, mirroring the pathological characteristics observed in humans (Senemaud et al., 2017; Fashandi et al., 2018; Golledge, 2019). The limitation of this model is that the formed AAA can appear in the suprarenal portion, which contrasts with the infrarenal location typically observed in human AAA. Furthermore, the primary pathology observed in this model is aortic dissection, whereas non-dissection AAA is predominantly observed in humans (Trachet et al., 2016).

### 5.3 Calcium chloride (CaCl_2_) application

CaCl_2_ is also frequently used for induction in AAA models. CaCl_2_ is directly applied to the infrarenal aorta (Chiou et al., 2001), the difficulty of the surgical procedure between the former two models. Moreover, the resulting aortic dilatation in the CaCl_2_ model is relatively mild. Similar to the elastase perfusion model, the CaCl_2_ model also involves acute injury and is not suitable for studies of long-term drug inhibition of AAA growth (Rd et al., 2001; Wang et al., 2014). In addition, the severity of AAA observed in this model is not as pronounced as in the two former models.

Every model possesses its own set of merits and demerits, with large animal models being comparatively less utilized within the realm of AAA research. AAA progresses more rapidly in existing animal models, whereas AAA in humans usually forms by slow progression. Consequently, the selection of animal models ought to prioritize the investigation of analogous pathogenic factors to human AAA, as well as the identification of potential therapeutic interventions.

## 6 Nanoparticles loaded with drugs for the treatment of AAA

**FIGURE 2:**
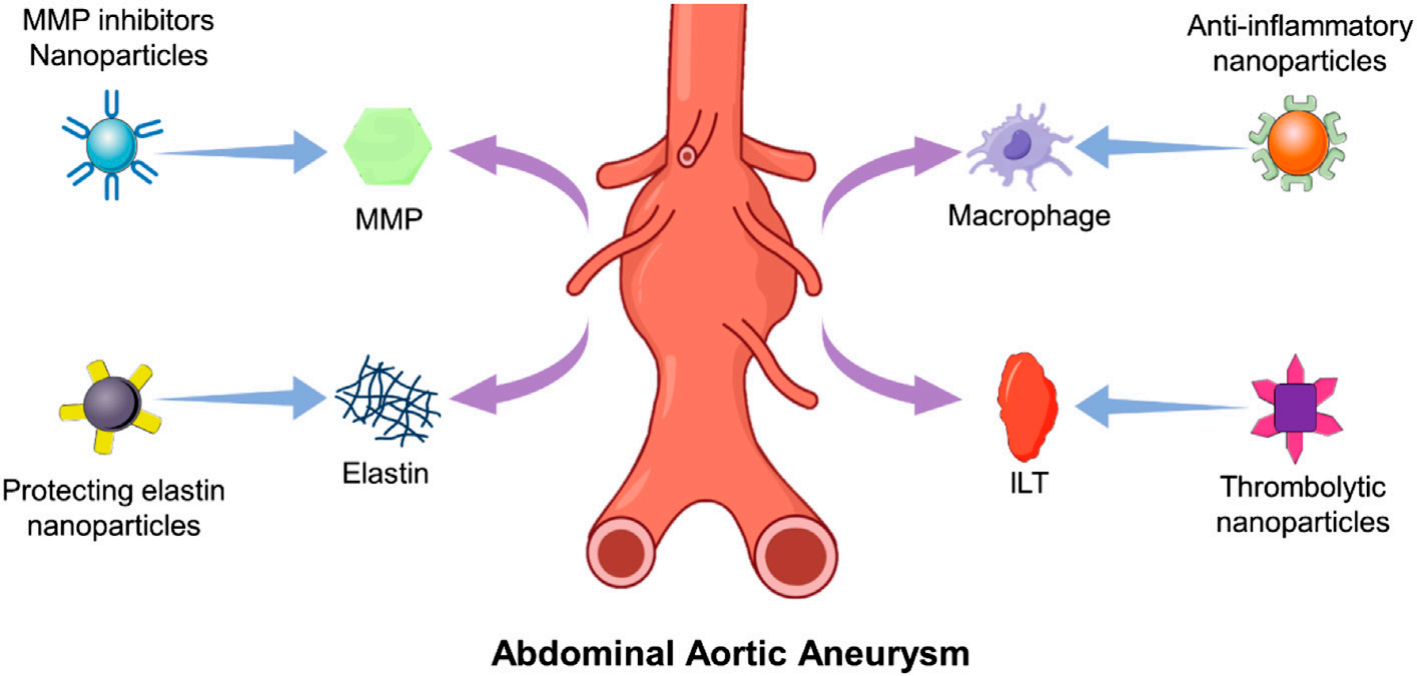
Drug-loaded nanoparticles target characteristic tissues and cells of AAA.

Currently, there is still no drug treatment for AAA in the clinic. The likely reason for this is the complex pathogenesis and biological structure of AAA, which prevents drugs from acting precisely at the site of injury to achieve effective concentrations. The development of engineered nanoparticles offers a new way to address the precise targeting of drugs (Hoshyar et al., 2016; Cheng et al., 2018; Zimmermann, 2018; Yin et al., 2021). The drug-loaded nanoparticles that have been used for AAA testing are summarized in Figure 2 and Table 2. We hope to find an emerging nanotechnology for innovative treatments for AAA.

**TABLE 2:** Nanoparticles loaded with drugs for the treatment of AAA.

Drugs	Nanoparticles	Model	Main finding	Refs
Batimastat	EL-PEG-PLA	CaCl_2_-induced rats	Inhibit MMP activity, Reduce elastin degradation	Sinha et al. (2014a)
Doxycycline	PLGA	Elastase-induced rats	Long-term drug release, inhibit MMP activity	(Sivaraman and Ramamurthi, 2013; Camardo et al., 2018)
	PEG-PLEA	Angiotensin II-induced mice	Enhance targeting, inhibit MMP activity	Sivaraman et al. (2017)
	PLGA + SPION			
PGG	EL-BSA	CaCl_2_-induced rats/mice	Reduce elastin degradation, regenerate elastin	Nosoudi et al. (2016a)
	EL-EDTA	CaCl_2_-induced rats/mice	Relief calcification, reduce elastin degradation, regenerate elastin	Nosoudi et al. (2016b), Dhital and Vyavahare (2020)
		Elastase-induced rats		
HA oligomer	PLGA	CaCl_2_-induced mice	Stable and slow release, increase elastic matrix, increase LOX activity	Sylvester et al. (2013)
TGF-β1	PLGA	Adult VSMCs within 3D collagen constructs	Increase elastic matrix	Venkataraman et al. (2016)
Rapamycin	OxbCD	CaCl_2_-induced rats	Anti-inflammatory, reduce oxidative stress response	Ding et al. (2005)
	PEG-b-PBLG	Elastase-induced rats	Anti-inflammatory	Shirasu et al. (2016)
Pitavastatin	PEG-PLys (FPBA)	Elastase-induced rats	Anti-inflammatory, Inhibit MMP-9	Fukuhara et al. (2020)
	PLGA	Angiotensin II-induced mice	reduce macrophage accumulation and MCP-1 expression	Katsuki et al. (2022)
tPA	PLGA	Fibrin clot of human blood, VSMCs of elastase-induced rats	Protect elastin matrix, Slow lyse ILT.	Sivaraman et al. (2016)

*AAA, abdominal aortic aneurysms; EL-PEG-PLA, elastin antibody-modified poly (ethylene glycol)-poly (lactic acid); BSA, bovine serum protein; PLGA, poly (lactic acid-glycolic acid); EDTA, ethylenediaminetetraacetic acid; PEG-b-PBLG, poly (ethylene glycol)-b-poly (γ-benzyl-L-glutamate); OxbCD, oxidation-responsive β-cyclodextrin material; PEG-Plys (FPBA), poly (ethylene glycol)-b-poly (l-lysine)-phenylboronic acid; ILT, intraluminal thrombosis; tPA, tissue-type plasminogen activator; PGG, pentagalloyl glucose; VSMCs, vascular smooth muscle cells; TGF-β1, transforming growth factor-β1; SPION, superparamagnetic iron oxide nanoparticles; HA, hyaluronic acid.

### 6.1 Nanoparticles loaded with MMP inhibitors

MMP inhibitors, which are a promising class of drugs for the treatment of AAA, have been shown to attenuate the degradation of ECM, but their use in the clinical treatment of AAA remains unsuccessful. The lack of control of dosage at the lesion resulting in toxicity (high doses) or ineffectiveness (low doses), together with poor water solubility and short circulation time, contributes to the unsatisfactory clinical outcome of drug-based therapy (Ding et al., 2005). By combining nanoparticles and MMP inhibitors, researchers have developed a new drug delivery system that allows aggregation of drug-laden nanoparticles with targeting capacity to the lesion (Bartoli et al., 2006; Yamawaki-Ogata et al., 2010; Nosoudi et al., 2015).

**FIGURE 3:**
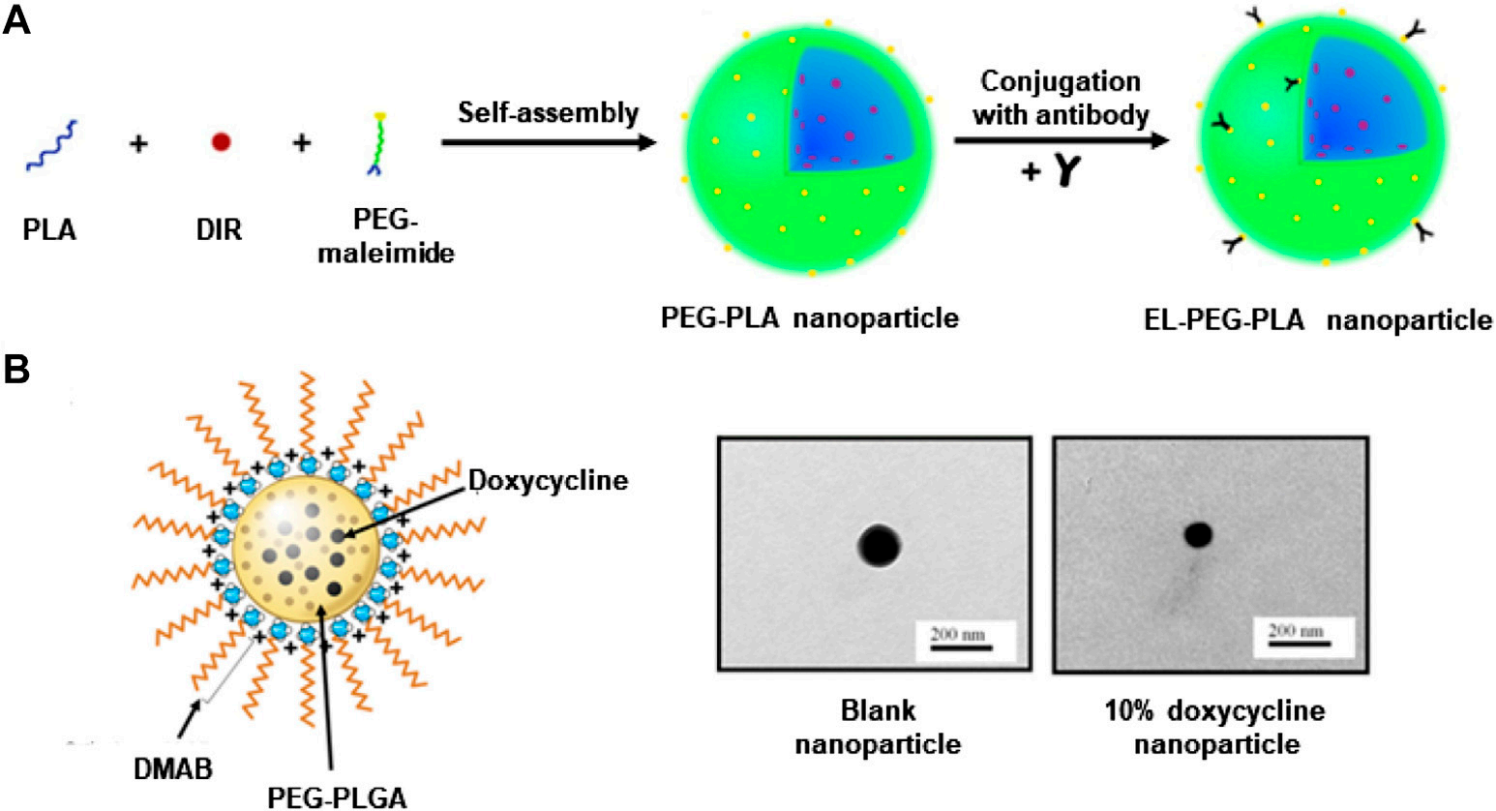
**(A)** Construct a schematic diagram of EL-PEG-PLA nanoparticles. **(B)** TEM and schematic diagram of PEG-PLGA nanoparticles loaded with doxycycline. Panel A reproduced from Ref. (Sinha et al., 2014a). Copyright 2014 Elsevier. Panel B reproduced from Ref. (Camardo et al., 2018). Copyright 2017 SpringerLink.

Batimastat is a small molecule MMP inhibitor. Sinha et al. developed an elastin antibody-modified poly (ethylene glycol)-poly (lactic acid) nanoparticles (EL-PEG-PLA) (Figure 3A) (Sinha et al., 2014a), loaded with Batimastat. In the CaCl_2_-induced rat AAA model, compared to free Batimastat, Batimastat-loaded EL-PEG-PLA nanoparticles effectively inhibited MMP activity, reduced elastin degradation, and prevented aneurysm growth (Sinha et al., 2014a; Nosoudi et al., 2015). These improvements are associated with the degradation of the fibres in the media layer of the AAA abdominal aorta, in which the amorphous elastin core proteins covered by fibres are exposed (Sakai et al., 1986; Sinha et al., 2014a). EL-PEG-PLA nanoparticles can target these proteins, aggregating at the lesion and improving bioavailability.

Another popular drug, doxycycline belongs to the group of tetracycline derivatives. It can inhibit the transcriptional process of genes through direct alignment with the catalytic site, thus inhibiting several MMP types (Liu et al., 2003). Even low-dose doxycycline administered systemically can cause significant side effects, so AAA patients do not benefit from clinical application (Ding et al., 2005; Sivaraman and Ramamurthi, 2013; Sylvester et al., 2013). Recent studies have found that localizing doxycycline via mini-osmotic pumps or peri-aortic foams can provide inhibition of MMP activity at very low doses (compared to systemic administration) (Bartoli et al., 2006; Yamawaki-Ogata et al., 2010). Therefore, targeting and slow release are necessary for doxycycline. Ramamurthi et al. reported that cationically functionalized poly (lactic acid-glycolic acid) (PLGA) nanoparticles containing doxycycline were able to release doxycycline locally over a long period to inhibit MMP activity and production (Sivaraman and Ramamurthi, 2013). In recent years, the same team has continued reporting several designs of doxycycline-loaded nanoparticles with a similar targeted therapeutic potential in an elastase-induced AAA rodent model (Sivaraman et al., 2017; Camardo et al., 2018). Particularly, one approach used a didodecyldimethyl ammonium bromide (DMAB) as a cationic surfactant to synthesize PEG-PLGA nanoparticles containing doxycycline through the double-emulsion technique, followed by PEGylation to extend the water solubility and circulating time. Fabricated nanoparticles were able to continuously release doxycycline at an effective dose for a long period (Figure 3B) (Camardo et al., 2018). In addition, another study used PLGA nanoparticles to encapsulate doxycycline and superparamagnetic iron oxide nanoparticles (SPION), which could be precisely directed to the lesion under the action of a magnetic field and release the drug to inhibit MMP activity (Sivaraman et al., 2017). These methods all provide continued release of doxycycline over 2 months, inhibiting MMP activity and promoting the preservation of the elastic matrix. May provide a non-surgical treatment option for AAA patients (Sivaraman et al., 2017; Camardo et al., 2018).

### 6.2 Nanoparticles protecting elastin layer

The main location of AAA injury is the elastic layer in the media of the aortic wall. This elastic layer is abundant in elastin. To effectively rehabilitate and safeguard the compromised aortic wall affected by AAA, it is crucial to impede any additional deterioration of elastin, stabilize the remaining elastic layer, and facilitate the regeneration of elastin.

Pentagalloyl glucose (PGG) is a naturally occurring polyphenolic compound with a high affinity for elastin, which protects, stabilizes, and promotes elastin regeneration (Sinha et al., 2014b; Nosoudi et al., 2016a). To utilize PGG for AAA therapeutics, Nosoudi et al. first prepared bovine serum protein (BSA) nanoparticles using the coacervation method, followed by the synthesis of PGG-loaded BSA nanoparticles via the double-emulsion and the incorporation of elastin antibody to increase targeting (EL-BSA). The *in vitro* results showed that the release of PGG continued for over 10 days. In CaCl_2_-induced AAA rats, reduced degradation of the elastic layer was observed compared to the control (nanoparticles without PGG), demonstrating a protective effect (Nosoudi et al., 2016a). The team noted during their experiments that this strategy was not effective for the removal of calcareous deposits. Therefore, the team further developed a new strategy for solving the calcification problem in a rat model by loading ethylenediaminetetraacetic acid (EDTA) onto EL-BSA nanoparticles via the reverse emulsion technique (EL-EDTA nanoparticles). In the same animal model, EL-EDTA nanoparticles were first used for 2 weeks to reduce existing calcification deposits, then EL-BSA nanoparticles were continued for 4 weeks, and histological analyses of the animals were performed at week 12, demonstrating that this combination therapy was effective in alleviating the calcification process (Nosoudi et al., 2016b). Dhital et al. similarly developed PGG-loaded nanoparticles and studied their effectiveness in an elastase-induced AAA model in rats. The findings of the study indicate that the examined nanoparticle exhibited inhibitory effects on MMP, while also demonstrating the ability to restore the elastic matrix, stabilize, and regenerate the elastic layer (Dhital and Vyavahare, 2020). These observed outcomes may be attributed to the potential of PGG to stimulate the production of lysyl oxidase (LOX), an enzyme that facilitates the cross-linking process of cohesive elastin. The cross-linking of collagen and elastin, catalyzed by LOX, plays a crucial role in maintaining the stability of the elastin layer (Sylvester et al., 2013).

Hyaluronic acid (HA) is a biocompatible polysaccharide widely found in the ECM, and studies have shown that the tight binding of HA to multifunctional proteoglycans plays a key role in elastogenesis (Bukhari et al., 2018). The fragment size of HA influences cellular function. Particularly, HA oligomers in the 4-mer to 6-mer (756–1,220 Da) range increase elastic fibril production and stability of the elastin matrix (Kothapalli et al., 2009). However, direct administration of HA oligomers has a short half-life in the blood and is unstable in the aneurysmal environment (Fraser et al., 1984; Isnard et al., 2001). To address this issue, Ramamurthi et al. applied a double-emulsion method to develop PLGA nanoparticles loaded with HA oligomers. Administrated nanoparticles could release HA oligomers stably over 30 days, increasing the elastin matrix synthesis and significantly enhancing the activity of LOX, which is important for the stabilization of elastin layer (Sylvester et al., 2013). The team also constructed PLGA nanoparticles loaded with transforming growth factor-β1 (TGF-β1). TGF-β1 regulates cell growth, proliferation, and apoptosis, and is also an elastogenic factor (Ghadami et al., 2000; Gacchina et al., 2011). Co-culture of these nanoparticles with human aortic SMC *in vitro* showed a significant increase in elastin content and matrix, suggesting that TGF-β1-based PLGA nanoparticles may also be a promising strategy for the treatment of AAA (Venkataraman et al., 2016).

### 6.3 Nanoparticles inhibiting inflammation

Inflammation is one of the main pathological characteristics of AAA. There are a variety of immune cells infiltrating the AAA vessel wall, in which macrophages are the main type (Sakalihasan et al., 2018; Zhao et al., 2021). Those macrophages are predominantly recruited to the external media of the aortic wall, leading to the release of proinflammatory cytokines. MMP and other proteases are produced, which ultimately lead to the degradation of elastin, collagen, and other proteins in the aortic wall, weakening the vessel wall and contributing to aneurysm development and progression (Raffort et al., 2017).

**FIGURE 4:**
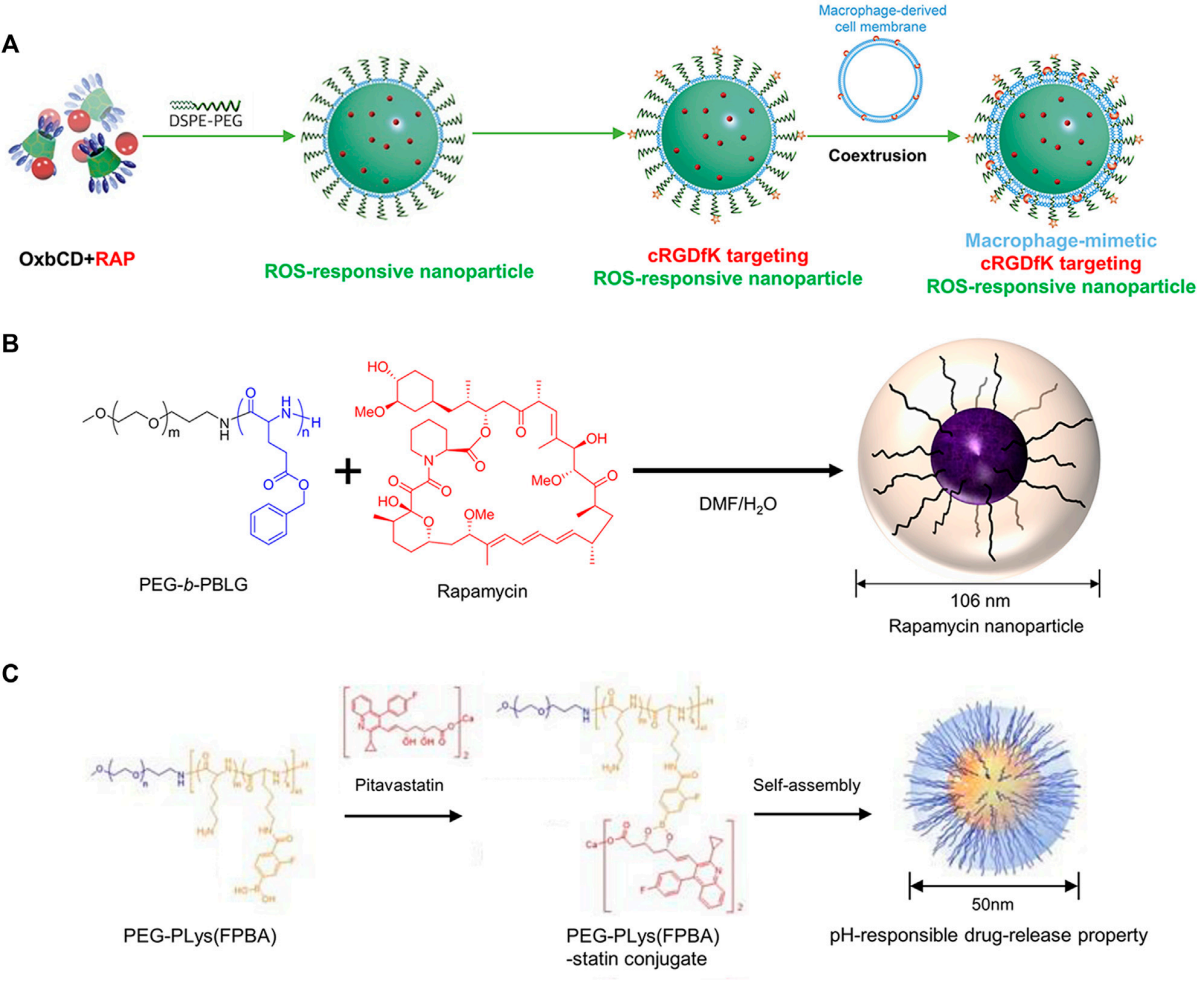
**(A)** The illustration of ROS-responsive targeting nanoparticles. **(B)** The illustration of mixing RAP and PEG-b-PBLG. **(C)** The illustration of mixing pitavastatin and PEG-PLys (FPBA). Panel A reprinted (adapted) from Ref. (Cheng et al., 2018). Copyright 2018 Elsevier. Panel B reproduced from Ref. (Shirasu et al., 2016). Copyright 2016 PLOS ONE. Panel C reproduced from Ref. (Fukuhara et al., 2020). Copyright 2020 MDPI.

Rapamycin (RAP) is a potent inhibitor of inflammation and also regulates pathophysiological effects such as cell growth, senescence, and atherosclerosis (Rouer et al., 2014). Limited effectiveness of RAP in reducing the progression of aneurysms was observed in many preclinical trials. The non-specific distribution of RAP, adverse effects after administration, and poor aqueous solubility have contributed to the disappointing outcomes (Lederle, 2013; Cheng et al., 2018). To improve the targeted delivery of RAP, various strategies have been developed, including ROX responsive targeting, since ROS overproduction ROS and oxidative stress are closely associated with AAA formation (Emeto et al., 2016). In one study, Cheng et al. constructed a hierarchical functionalization strategy to develop ROS-responsive atheroma-targeting nanoparticles (oxidation-responsive β-cyclodextrin material (OxbCD)) to enable the delivery of RAP to treat AAA (Figure 4A). The cRGDfK (a cyclopeptide ligand) combined with the nanoparticles, and then coextruded pre-prepared macrophage cell membrane vesicles through a 200 nm polycarbonate membrane and coated onto the core of the nanoparticles to complete the macrophage membrane modification. The fabricated nanoparticles exhibited excellent aneurysm targeting ability due to the specific binding between ligands and macrophage membranes. More importantly, the effective aggregation of the nanoparticles at the lesion resulted in sufficient drug dosage with anti-inflammatory effects in CaCl_2_-induced AAA rats (Cheng et al., 2018). In another study, Shirasu et al. delivered RAP via Poly (ethylene glycol)-b-poly (γ-benzyl L-glutamate) nanoparticles (PEG-b-PBLG) (Figure 4B). In an elastase-induced AAA rat model, enhanced uptake of nanoparticles by macrophages at the AAA lesion was observed. The nanoparticles can be loaded with a low dose (0.1 mg/kg) of RAP to achieve relief of aneurysm dilatation, whereas the same dose of free RAP does not affect AAA. PEG-b-PBLG nanoparticles loaded with RAP resulted in a reduction of inflammatory cytokine production in the aneurysm wall and inhibited MMP, which contributed to the construction of a drug for the prevention and treatment of AAA (Shirasu et al., 2016).

Besides RAP, statins are inhibitors of hydroxymethylglutaryl-coenzyme A (HMG-CoA) reductase, which has been widely used for AS treatment, because of their lipid-lowering capacity, anti-inflammatory, and anti-atherosclerotic properties. Many preclinical studies have demonstrated that statins can inhibit the progression of AAA (Steinmetz et al., 2005). However, there is a study claiming that statins are not effective in aneurysms in humans (Itoga et al., 2018), this study was based on oral administration, so it does not negate the possibility of *in vivo* treatment with nanoparticles loaded with statins. For instance, Fukuhara et al. fabricated poly (ethylene glycol)-b-poly (l-lysine)-phenylboronic acid (PEG-PLys (FPBA)) nanoparticles that contained pitavastatin (Figure 4C). In elastase-induced AAA rats, nanoparticles effectively inhibited AAA expansion by inhibiting macrophage and MMP-9 after intravenous injection, compared to free pitavastatin (Fukuhara et al., 2020). Similarly, Katsuki et al. used PLGA as a drug carrier to synthesize nanoparticles loaded with pitavastatin. In an angiotensin II-induced AAA mice model, intravenous administration of these drug-loaded nanoparticles was found to reduce macrophage accumulation and monocyte chemotactic protein-1 (MCP-1) expression, contributing to the inhibition of AAA formation (Katsuki et al., 2022). Excitingly, the nanoparticles constructed in this study have been tested in Phase I and Phase IIa clinical trials in healthy subjects and patients with severe limb ischemia (clinical trial registry identifier: UMIN000014940 and UMIN000019189). Notably, no significant adverse effects were observed, prompting the initiation of a future large-scale clinical trial. This provides a new strategy for the clinical translation of nanoparticle therapy for AAA (Katsuki et al., 2022). Current drug studies of statins for the prevention and treatment of AAA are limited to pitavastatin, which may be related to its strong anti-inflammatory effect (Aoki et al., 2009).

### 6.4 Nanoparticles loaded with thrombolytic drugs

Tissue-type plasminogen activator (tPA) is a thrombolytic drug that converts plasminogen to plasmin in the blood, thereby rapidly resolving cardiovascular and cerebrovascular thrombotic events, restoring blood supply to organs, and reducing mortality (Fugate and Rabinstein, 2014). Three-quarters of AAA patients have intraluminal thrombosis (ILT) (Sun et al., 2009). However, in AAA, rapid thrombolysis is undesirable because high levels of fibrinolytic enzymes enhance MMP expression, which promotes ECM degradation. In addition, rapid thrombus lysis exposes proteases and inflammatory cells in the ILT to the post-thrombolytic lumen of the aortic wall, resulting in loss of (bio)mechanical barrier of the damaged aortic wall (Kazi et al., 2003; Sun et al., 2009). Slow and controlled thrombolysis, together with regulation of fibrinolytic enzyme production may slow AAA progression and rupture (Sivaraman et al., 2016). As a result, tPA could have the potential to alleviate the AAA. To prove the potential of tPA in AAA management, Sivaraman et al. fabricated PLGA nanoparticles loaded with tPA. Synthesized nanoparticles could slowly lyse fibrin clots *in vitro* without inducing proteolytic damage. The nanoparticles were further surface functionalized with cationic amphiphiles to bind to anionic fibrin clots. The results showed that slowly released tPA contributed to the gradual dissolution of ILT, which is beneficial to protect the elastic matrix within the adjacent AAA wall from disintegration. Furthermore, the porous structure of ILT resulting from tPA facilitated the penetration of therapeutic agents from the circulatory system into the AAA wall (Sivaraman et al., 2016). *In vivo*, the environment is more complex and the level of inflammatory activity is significantly higher than *in vitro*. Platelets and erythrocytes in the ILT may affect nanoparticle binding and thrombolytic effects, without achieving the desired penetration and thrombolysis.

### 6.5 Nanoparticles and stent-grafts

**FIGURE 5:**
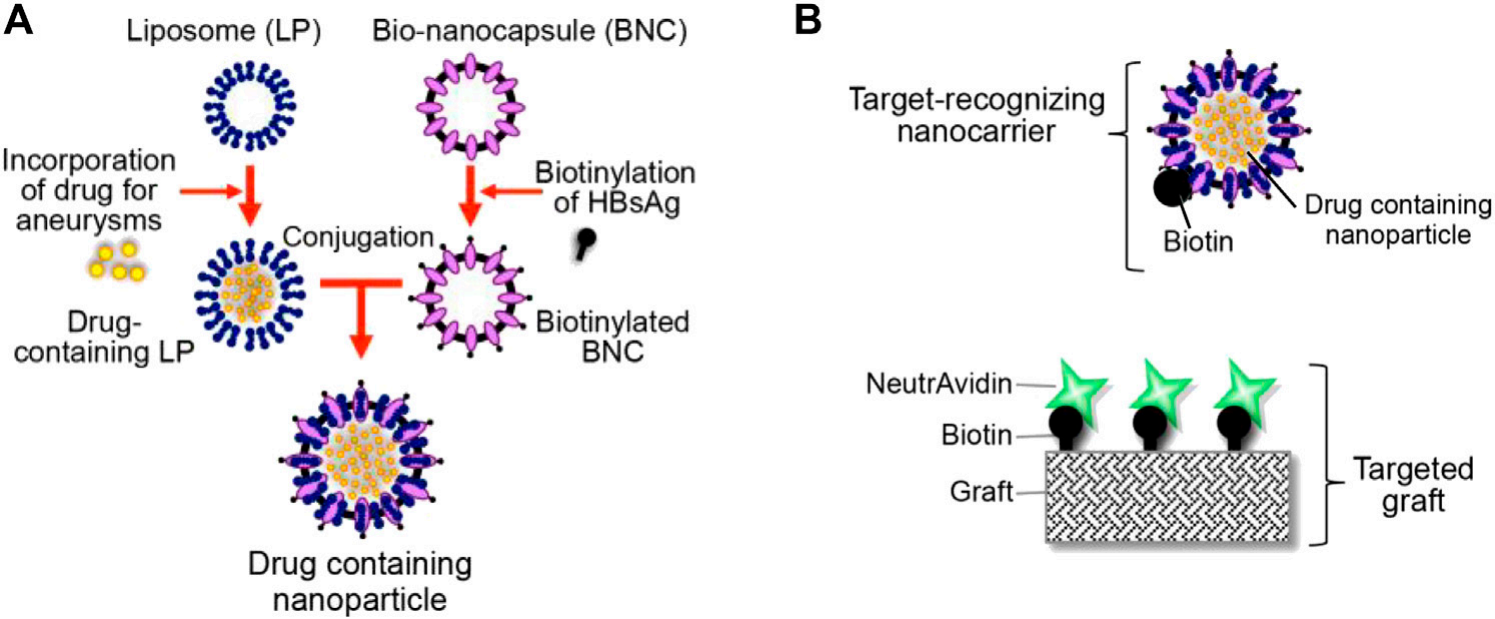
**(A)** Diagram shows the liposome-based biotinylated drug-loaded nanoparticle. **(B)** Diagram shows the targeted graft coated with biotin–neutravidin complexes. Panel reprinted (adapted) from Ref. (Yoshimura et al., 2020). Copyright 2020 MDPI.

After EVAR surgery for AAA patients, the stent-grafts can open new physical channels for main arterial blood flow and protect the aortic wall from further dilation, but they cannot treat the damage to the vessel wall that has already developed. his can predispose to common complications such as endoleak and aneurysm rupture, which need to be addressed. Drug-coated stent-grafts have been developed to provide local drug therapy, aiming to reduce the potential complications. That being said, the short drug release time at the lesion negatively impacts the therapeutic effect. To solve this issue, Yoshimura et al. have developed a novel drug-targeted stent-graft delivery system with repeated drug administration potential (Yoshimura et al., 2020). The system had two components, a liposome-based biotinylated nanoparticle and a stent-graft coated with biotinylated poly (2-hydroxyethyl methacrylate) (pHEMA) (Figure 5). The drug-loaded nanoparticles can be targeted to the stent-grafts through non-covalent interactions. After further implanted pHEMA stent-grafts into mice, they demonstrated drug release at the target site following systemic injection of drug-laden nanoparticles (Yoshimura et al., 2020). The popularity of stent-grafts provides a new strategy for continued treatment after EVAR.

## 7 EVs-based nanotechnology for AAA

As mediators of intercellular communication, EVs are heavily involved in the physiological and pathological processes of many cardiovascular diseases (Walker et al., 2019). Many studies have found that EVs are tissue chemotactic. They can deliver biomolecules to recipient cells, and exhibit a good safety profile, which makes them ideal drug delivery vehicles (Dai et al., 2008). In addition, EVs can be harvested from cell culture conditions or biological tissues by extrusion, ultrasound, and other isolate methods. More importantly, they are also very stable in long-term cryopreservation and transportation (Yu et al., 2014; Witwer and Wolfram, 2021).

To date, several studies have explored the potential of EVs in AAA management. For example, Sajeesh et al. found that EVs appear to balance the proteolytic environment of aortic lesions and enhance TIMP activity, a natural inhibitor of MMP, with potential therapeutic value in the early treatment of AAA (Sajeesh et al., 2020). Additional studies have also found that microRNAs (miRNAs) in EVs had different effects on AAA development. Mesenchymal stem cell (MSC)-derived EVs could resolve the inflammatory response and aneurysm progression in AAA through miR-147 (Spinosa et al., 2018). Meanwhile, MiR-106a within EVs could downregulate TIMP-2 expression, restore the production of MMP, and accelerate VSMC apoptosis and ECM degradation (Han et al., 2020), leading to the occurrence of AAA. In addition, overexpression of miR-29b appears to promote AAA progression in rat models. As a result, loading the desirable cargo within EVs could be a promising strategy for AAA treatment.

**FIGURE 6:**
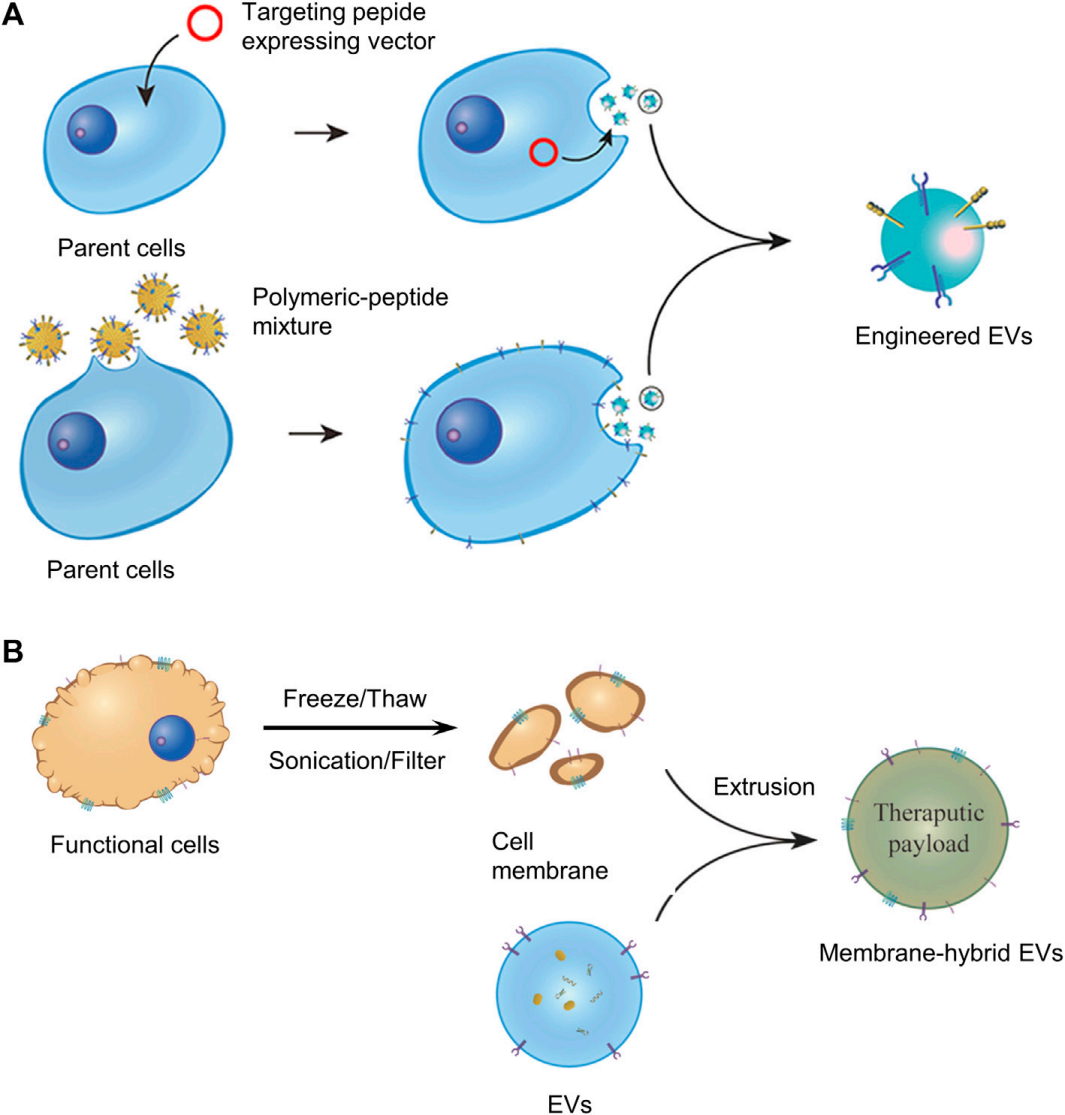
**(A)** Display technology for the construction of engineered EVs. **(B)** Cell membrane fusion technology for the construction of engineered EVs. Panel reprinted (adapted) from Ref. (Lu et al., 2022). Copyright 2022 Frontiers.

Despite the many advantages of naturally secreted EVs, their use in therapy is further hampered by disadvantages, including lack of targeting, toxic reactions at high doses, and rapid clearance *in vivo* by the mononuclear phagocytic system (Wiklander et al., 2015; Wan et al., 2020; Witwer and Wolfram, 2021). To address these issues, natural EVs can be further modified by tissue engineering approaches. For instance, adding targeting ligands (EV display technology), and cell membrane fusion techniques could improve their targeting and circulatory delivery efficiency (Luk and Zhang, 2015; Witwer and Wolfram, 2021; Lu et al., 2022), as shown in Figure 6.

A method of EV display technology is to add targeting ligands by transfecting parent cells with fusion genes for targeting peptides and EV membrane proteins. Overexpression of fusion proteins on the surface of EV membranes moves engineered EVs directly toward the target area. However, there are many problems with EV display technology, for example, the process is very labor-intensive and time-consuming, and the added functional membrane proteins are sometimes degraded by proteases of cells or body fluids, losing their targeting properties. Enhancing the stability and persistence of the targeting peptide is very meaningful for the further development of EV display technology (Lu et al., 2022). The cell membrane fusion technology is also getting more and more attention, which can fuse EV membranes with the cell membranes of many kinds of cells in biomedical applications.

## 8 Conclusions and future perspective

Presently, the clinical management of AAA predominantly revolves around surgical intervention, specifically for individuals at risk of AAA rupture and patients with large AAA. However, no pharmacological alternatives exist for patients who are unable to undergo surgery, individuals with small AAA, and those experiencing ongoing AAA progression. Several studies have reported the advantages of drug-loaded engineered nanoparticles in the treatment of AAA, including the nanoparticles to increase the slow release and targeting of drugs, which can be localized in AAA at lower drug concentrations, showing good efficacy and safety.

However, challenges remain for further clinical translation. Firstly, some materials have high costs and long lead times to construct nanoparticles, and some show potential immunogenicity, or the constructed nanoparticles are so small that they can be quickly cleared *in vivo*. Secondly, when using antibody proteins to increase nanoparticle targeting, these antibodies are prone to denaturation during the construction process and may be degraded by a variety of enzymes *in vivo*, with loss of targeting. Finally, preclinical studies on nanoparticle drug delivery for AAA mainly focus on rodent models, which have shown exciting results. However, using only a single model cannot perfectly replace the pathogenic environment of AAA patients (e.g., pressure, flow rate, and composition of internal circulation), and further validation in multiple models is needed before entering clinical practice.

In the future, with the continuous innovation of nanotechnology, the maturation of gene editing technology, and the emergence of new natural nanomaterials such as EVs. Improving the targeting potential, circulation time, and controllable release of drugs by constructing engineered nanoparticles will become more diverse, which will provide new avenues for the next-generation of developing nano-loaded therapeutic AAA.
